# Conducting multiple mini-interviews in the midst of COVID-19 pandemic

**DOI:** 10.1080/10872981.2021.1891610

**Published:** 2021-02-23

**Authors:** Kenneth Yy Kok, Lie Chen, Fazean Irdayati Idris, Nuramalina H Mumin, Hazim Ghani, Ihsan Nazurah Zulkipli, Mei Ann Lim

**Affiliations:** Pengiran Anak Puteri Rashidah Sa’adatul Bolkiah Institute of Health Sciences, Universiti Brunei Darussalam, Bandar Seri Begawan, Brunei Darussalam

**Keywords:** Multiple mini-interview, internet, selection, medicine programme

## Abstract

Multiple mini-interview (MMI) is a ‘multiple sample-based’ approach comprising multiple focused encounters intended to access and assess a range of attributes in order to gain more objectively multiple impressions of an applicant’s interpersonal skills, thoughtfulness and general demeanour. It is designed to focus on four domains that are not considered to be comprehensive, but are considered to be vital for a successful career in the health sciences: critical thinking, ethical decision making, communication and knowledge of the healthcare system. Traditionally, the MMI is conducted face-to-face, but with COVID-19 pandemic and the implementation of social distancing measures, no onsite or campus teaching, banning of mass gatherings and cancellation of face-to-face interviews, Pengiran Anak Puteri Rashidah Sa’adatul Bolkiah Institute of Health Sciences at Universiti Brunei Darussalam explored the feasibility of conducting MMI through virtual means. This report provides an account of our experience in conducting internet-MMI for the selection of new applicants into the August 2020 cohort of the Medicine programme. We also aimed to determine whether the scores derived from internet-MMI were reliable and equivalent to the scores derived from traditional MMI.

## Introduction

The multiple mini-interview (MMI) was first introduced by Eva *et al*. in 2002 to assess the non-academic attributes of candidates who applied to the undergraduate medical degree (MD) programme at McMaster University, Canada [1]. MMI is a ‘multiple sample-based’ approach comprising multiple focused encounters intended to access and assess a range of attributes in order to gain more objectively multiple impressions of an applicant’s interpersonal skills, thoughtfulness and general demeanour [2]. This approach has been shown to have reasonable feasibility, acceptability, validity and reliability in the selection of candidates into undergraduate medicine programmes [3], and has been adopted by other undergraduate health programmes such as dentistry, pharmacy, nursing, physiotherapy and veterinary science, as well as by postgraduate programmes [4].

MMI is designed to focus on four domains that are not considered to be comprehensive, but are considered to be vital for a successful career in the health sciences: critical thinking, ethical decision making, communication and knowledge of the healthcare system [1]. There are a number of variations on how MMI is conducted. Traditionally, the MMI is conducted face-to-face with a circuit of 5 to 12 stations. Each station comprises an encounter with one interviewer, which lasts from 5 to 8 minutes, and a turnaround time between the stations. The interview usually includes 4 to 5 prompts related to the question scenario that the interviewer can use if the candidate does not provide responses spontaneously. Each candidate is rated using either a Likert scale or a normative scoring rubric system. In addition, each candidate is given a rating based on global impression of their interview performance [5].

Since its inception in 2006, Pengiran Anak Puteri Rashidah Sa’adatul Bolkiah (PAPRSB) Institute of Health Sciences at Universiti Brunei Darussalam (UBD) has incorporated MMI as the standard admission process, along with satisfactory pre-university final academic grades to select candidates into the Medicine programme. The MMI conducted in PAPRSB Institute of Health Sciences involves a structured interview where candidates progress through a circuit of 10 stations with individual interviewers. Each station assesses a specific skill or knowledge in domains such as personal qualities, presentation skills, thought organisation, self-reflection, critical thinking, patient confidentiality, ethical decision-making, awareness of current medical and health issues, communication skill and knowledge of health system. Each station lasts for 5 minutes with 2 minutes changeover time between stations. The candidate is rated using an analytic rubrics scoring system with scores out of 10 for each station. Each candidate is scored out of 100 marks for the whole MMI. In addition, the candidate is also given a score out of 5 based on the interviewer’s opinion on the global impression of the candidate’s performance for each of the station and their suitability for the Medicine programme.

On 11 March 2020, as the numbers of confirmed cases and deaths due to COVID-19 continued to rise globally since its emergence in Wuhan, China in December 2019, the World Health Organization declared the COVID-19 outbreak a pandemic [6]. Since the declaration of COVID-19 pandemic, and with the advice from the Ministry of Health, Brunei Darussalam, UBD has implemented social distancing measures, including no onsite or campus teaching, banning of mass gatherings and cancellation of face-to-face interviews. Due to these restrictions, the PAPRSB Institute of Health Sciences Medicine programme team had to consider other viable ways to conduct the high-stakes MMI for the selection of new applicants into the August 2020 cohort of the Medicine programme. The team decided to explore the feasibility of conducting the MMI through virtual means.

### Internet-MMI process in PAPRSB institute of health sciences

In March 2020, 48 candidates were shortlisted for selection into the August 2020 Medicine programme, but 1 declined to be interviewed. The decision to conduct internet-MMI (iMMI) was made a day before the interview, using *Zoom* (Zoom Video Communications, 2011), a digital cloud-based video platform with the support of *WhatsApp Messenger* (WhatsApp Inc., 2009), an instant messaging service for easy communication with candidates. All candidates were informed via email and short message service (SMS) or *WhatsApp Messenger* regarding the official decision to conduct the iMMI and they were instructed to install the *Zoom* application into their personal electronic devices (computers or smartphones). Meeting ID or Personal Link Name for joining the *Zoom* meeting were given to the candidates during the iMMI session via *Zoom* chat.

The iMMI was arranged into three circuits. Each circuit included four 7-minute stations with a 3-minute changeover time between stations. The questions across the four stations were designed to assess each candidate’s level of critical thinking, ethics and moral decision making, awareness of current medical and health issues and self-reflection. Each station comprised an interviewer with a laptop or desktop computer installed with *Zoom* connection (with audio and video set up) either in the interviewer’s own office or residence. Interviewers were instructed to enable *Zoom*’s Waiting Room feature. Three staff members acted as coordinators, one for each circuit. Approximately 30 minutes prior to the iMMI, candidates were contacted via *Zoom* and SMS by the coordinators to check for stable internet connection and to verify their identity. The candidates remained connected in a virtual holding room while waiting to be invited to the iMMI stations. While waiting in the virtual holding room, the candidates were briefed by the coordinator on the iMMI process and instructed to rename their screen name to assigned codes to ensure anonymity.

The iMMI started as soon as the candidate clicked on ‘Join’ and entered the interviewer’s *Zoom* Meeting ID or Personal Link Name, which was provided by the coordinator. Once the interviewer had introduced themselves, they sent the question scenario to the candidate using the *Zoom* ‘Share Screen’ option. The candidate was given 2 minutes to study the question scenario, in the form of a passage or a picture, which was followed by a 5-minute discussion between the interviewer and candidate regarding the scenario. On completion of the iMMI station, the candidate was requested to ‘Leave Meeting’, and was then contacted by the coordinator to join the next MMI station by providing them with the next interviewer’s Meeting ID or Personal Link Name via *Zoom* chat. The candidate moved from one station to the next until they completed all four stations. It was important that the interviewer’s Waiting Room feature was enabled. During each step of the iMMI process, all the interviewers were kept in contact via a *WhatsApp* group chat specifically created for the interviewers and coordinators only. In the interests of examination security, candidates were corralled before and after the interview into the coordinator’s virtual holding room to ensure no contact between candidates who had taken the iMMI and those who were about to take it. The whole iMMI process took about 3 hours with the three circuits conducted simultaneously, and with the involvement of three coordinators, 12 interviewers and 47 candidates.

## Outcomes of internet-MMI

All 47 candidates completed the iMMI. Each station was scored out of 10 marks. The mean and median scores for all the candidates for all four stations were 26.9 and 26.5 respectively (range 21.0–36.0). After converting the individual score to percentage, the mean and median percentage scores for all candidates were 67.3 and 66.3 respectively with SD 8.8 (range 52.5% – 90.0%). The Cronbach Alphas for the four individual stations ranged from 0.695 to 0.710 (with the internal consistencies of the iMMI station within the acceptable range).

After the iMMI, both the interviewers and candidates were contacted by the authors via *WhatsApp* and email to share their experience on the iMMI, and whether or not they had encountered any technical problems before and during the process. The interviewers reported no major interruptions during the iMMI process, and were able to simultaneously managed both *Zoom* and *WhatsApp* deftly. Coordinators reported that a number of candidates were initially unable to join the interviewer’s *Zoom* meeting after entering the interviewer’s Meeting ID. The problem was easily solved by restarting the *Zoom* app or their electronic devices. A number of candidates reported poor audio quality using their laptops, but the switch to their smartphones improved the audio quality. The majority of the candidates found the iMMI experience good and not as complicated as previously thought, even though it was the first time that they attended an online interview.

In order to determine whether the scores derived from the iMMI were equivalent to and as reliable as those scores derived from the traditional face-to-face MMI, we set out to compare the scores of the iMMI with the scores derived from candidates who underwent the traditional MMI last year before the pandemic. In 2019, a total number of 43 candidates underwent the traditional MMI. The mean and median percentage scores for the traditional MMI candidates were 69.1 and 69.0 respectively with SD 6.6 (range 55.5% – 84.0%). There was no significant statistical difference between scores of the candidates who underwent iMMI and the candidates who had traditional face-to-face MMI (Independent Sample T-test, *p = 0.285*).

## Discussion

MMI has been important part of PAPRSB Institute of Health Sciences selection process for the admission of students for the Medicine programme. When COVID-19 pandemic was declared and with the implementation of social distancing measures, including banning of mass gatherings and cancellation of face-to-face interviews, our Medicine team decided to resort to virtual means to conduct the MMI of the high-stakes admission process. Our experience has shown that it was feasible to conduct the MMI using *Zoom* and *WhatsApp* applications. We have demonstrated evidence for the equivalence of the iMMI and the traditional MMI. Despite the smaller number of stations in iMMI, the average score obtained from candidates in the iMMI did not significantly differ from candidates who underwent traditional MMI.

Web-based panel or personal interviews have been utilized by medical schools to assess applicants [7]. However, there has only been one reported account of an internet-based MMI [8]. In 2011, Tiller et al. at the University of Sydney, Australia successfully conducted an MMI through *Skype* to assess international applicants for their medical and dental graduate programmes. The authors showed that the interviewers were able to make valid and reliable decisions about candidates through the internet-based MMI that was acceptable to participants, producing comparable results to the traditional in-person MMI with a saving of resources [8]. Our own experience showed that internet-based MMI using the *Zoom* platform supplemented with *WhatsApp Messenger* could be utilized to conduct the selection of new applicants into the Medicine programme, with valid and comparable scores to the traditional face-to-face MMI. Our findings, which are in agreement with that of Tiller et al. [8], showed that internet-MMI was acceptable to both interviewers and candidates and produced scores that were equivalent to the traditional MMI. The majority of our candidates had also found the iMMI experience good even though it was the first time that they attended a virtual interview.

## Conclusions

Our experience showed that good teamwork, skillful coordination and a reliable high-speed internet connection are essential to conduct a successful internet-based MMI. Studies with larger sample of candidates and more iMMI stations, together with studies which compare directly the in-person MMI and internet-based MMI are required to validate the use of iMMI in selecting candidates for health science programmes. Nonetheless, our experience showed that it is feasible to conduct internet-based MMI and the interviewers were able to make valid and reliable decisions about the candidates through the internet-based MMI during the COVID-19 pandemic when social distancing is mandatory. It also suggests that virtual or online interviews may be an effective method for the admissions procedure without the need for physical presence or face-to-face interviews.
